# Branched‐Chain Amino Acids and Di‐Alanine Supplementation Attenuates Muscle Atrophy in a Murine Model of Cancer Cachexia

**DOI:** 10.1111/apha.70067

**Published:** 2025-05-31

**Authors:** Mayra Colardo, Noemi Martella, Michela Varone, Daniele Pensabene, Giuseppina Caretti, Gianluca Bianchini, Andrea Aramini, Marco Segatto

**Affiliations:** ^1^ Department of Biosciences and Territory University of Molise Pesche Italy; ^2^ Department of Biosciences University of Milan Milan Italy; ^3^ Research & Early Development Dompé farmaceutici S.p.A. L'Aquila Italy

**Keywords:** BCAA, dietary supplements, muscle wasting, nutraceuticals, protein catabolism, skeletal muscle

## Abstract

**Aim:**

Cancer cachexia is a severe metabolic disorder leading to skeletal muscle atrophy. Muscle wasting is a major clinical problem in cachectic patients, as it limits the efficacy of chemotherapeutic treatments and worsens quality of life. Nutritional support based on branched‐chain amino acids (BCAA) has been shown to be a promising approach to counteract cachexia‐induced muscle atrophy, but its efficacy is still debated. Furthermore, the putative role of di‐alanine (Di‐Ala) supplementation has yet to be evaluated. The present study therefore sought to assess whether BCAA supplementation, alone or in combination with a Di‐Ala peptide, could attenuate muscle wasting in a preclinical model of cancer cachexia.

**Methods:**

To this end, C26 tumor‐bearing mice were administered BCAA supplementation, with or without Di‐Ala. Body and muscle weights, as well as molecular, biochemical, and morphological analysis, were carried out to characterize prospective changes of markers involved in cachexia and muscle atrophy.

**Results:**

The main findings revealed that BCAA supplementation effectively prevented body weight loss and muscle atrophy. Of note, Di‐Ala significantly amplified the effects of BCAA. These phenomena were found to be mediated by the suppression of pathways involved in protein catabolism.

**Conclusions:**

Collectively, these results highlight that innovative formulations containing Di‐Ala, capable of increasing BCAA bioavailability, may be efficacious in counteracting muscle atrophy, especially during mild‐to‐moderate cancer cachexia.

## 1 Introduction

Muscle atrophy is a condition characterized by a functional loss of skeletal muscle mass associated with a reduction in myofiber volume [1]. Muscle atrophy occurs not only during aging due to the age‐dependent loss of homeostasis, but is also frequently related to a number of pathological contexts, such as cancer cachexia [1, 2]. The molecular mechanisms responsible for muscle atrophy in cancer cachexia are analogous to those that mediate other muscle wasting‐related conditions. Indeed, muscle function is affected by the turnover of contractile proteins, with the production of new myofibrils and protein degradation depending on a delicate balance that is strongly regulated by diverse signaling pathways. These transduction cascades are activated in response to mechanical stress, physical activity, nutrient availability, and growth factors [1]. Consequently, the mechanisms that culminate in atrophy are generated by an imbalance in the regulatory processes governing protein anabolism and catabolism within muscle tissue. Specifically, the loss of muscle mass in cancer cachexia is caused by a dramatic augmentation of protein degradation via autophagy and the ubiquitin–proteasome system (UPS), as well as a reduction in protein biosynthesis [1, 2]. Notably, the imbalance between anabolic and catabolic processes related to protein metabolism is predominantly driven by systemic inflammation [3].

Muscle wasting is a major clinical problem in cancer patients. In fact, the loss of muscle mass not only hinders patients' mobility but also limits the effectiveness of therapeutic approaches. The existing relationship between muscle atrophy and clinical outcomes is not surprising: skeletal muscle should no longer be considered a simple tissue devoted to locomotor function, as it produces and releases a variety of signaling molecules (i.e., myokines), thus acting as a hormonal tissue that contributes to the overall homeostasis [4]. As indicated by the extant literature, it is evident that muscle atrophy is a condition with a high social, clinical, and economic impact [5]. Consequently, it is attracting the interest of clinicians, researchers, patients, and society at large.

In recent years, a plethora of therapeutic interventions have been explored with the objective of counteracting muscle mass loss, both in the preclinical and clinical studies. The most investigated approaches involve the administration of growth factors and molecules with anabolic activity, treatment with anti‐inflammatory compounds, electrical stimulation, and, when possible, exercise and physiotherapeutic rehabilitation [6, 7, 8, 9, 10].

Nutritional support, specifically in terms of protein component, also represents a possible therapeutic strategy of relevant interest. The nutritional interventions under investigation are numerous, and particular attention has been addressed to dietary supplementation with branched‐chain amino acids (BCAA). BCAA are a group of three essential amino acids (leucine, isoleucine, and valine) that strongly stimulate protein synthesis [11]. Additionally, other evidence has suggested that these amino acids are also involved in the suppression of pathways that mediate protein catabolism [12]. In support of this, nutritional supplementation with BCAA was shown to counteract muscle atrophy in several pathophysiological contexts, including cancer cachexia [13].

Despite this evidence, the information available in the literature still remains limited, and the presence of contradictory data does not clearly define the potential contribution of dietary supplementation with BCAA in the prevention of muscle atrophy [14, 15, 16]. Furthermore, no experimental work has so far highlighted the possible benefit of dietary supplementation based on a combined BCAA and alanine (Ala) formulation in muscle atrophy during cancer cachexia.

In this respect, the degradation rate of BCAA is closely associated with the availability of Ala (Ala) and glutamine (Gln), two highly proteinogenic amino acids [17]. One of the main events triggered by a BCAA‐enriched diet is the increase in circulating levels of Ala and Gln, suggesting that many effects of BCAA are mediated by Ala and Gln [17, 18]. Several lines of evidence indicate that BCAA are the main source of nitrogen required for Ala synthesis, and clinical studies have shown that more than 60% of plasma Ala is derived from *de novo* synthesis following dietary supplementation with BCAA [17, 19]. Several preclinical studies have suggested that Ala administration (alone or in combination with other compounds) significantly reduces BCAA catabolism, improving locomotor function and endurance in rodents undergoing physical activity [17, 20, 21]. Furthermore, recent data demonstrate the efficacy of a new oral mixture of BCAA associated with the dipeptide l‐Alanyl‐l‐Alanine (Di‐Ala) in counteracting muscle atrophy in conditions other than cancer cachexia, such as muscle disuse or aging‐associated sarcopenia [22, 23]. The enhanced bioavailability of Ala, resulting from Di‐Ala administration, is attributable to the increased rate of intestinal uptake and absorption of dipeptides in comparison to free amino acids [24, 25]. This phenomenon may consequently lead to an augmentation in BCAA bioavailability. In addition, Di‐Ala has been demonstrated to be a highly efficacious activator of the human intestinal oligopeptide transporter (PEPT1), which plays a pivotal role in the uptake of oligopeptides across the enterocytes' brush border membrane [26].

In the light of these notions, the objective of this study was to evaluate the potential protective effects of dietary supplementation with BCAA, administered alone or in combination with the dipeptide Di‐Ala, in the prevention of muscle atrophy in a preclinical mouse model of cancer cachexia.

## 2 Materials and Methods

### 2.1 Animals

Three‐month‐old male BALB/c mice were housed in groups of five and maintained under controlled temperature (20°C ± 1°C), humidity (55% ± 10%), and lighting (12/12 h light cycle with lights on at 07:30). Food and water were provided ad libitum. All mice were quarantined for 1 week prior to the experiments. All procedures involving animal care or treatment were approved by the Italian Ministry of Health (authorization n. 169/2022‐PR) and were performed in accordance with the guidelines of the Italian Ministry of Health (according to Legislative Decree 116/92) and Directive 2010/63/EU of the European Parliament and of the Council of 22 September 2010 on the protection of animals used for scientific purposes. Tumor‐bearing mice received 500 000 C26 colon cancer cells in PBS by dorsal subcutaneous (s.c.) injection. Control mice were injected with PBS. The animals were then randomized and divided into four groups: control (mice without tumor cell inoculation), C26 (C26‐bearing mice treated with vehicle), C26+A (C26‐bearing mice treated with BCAA), and C26+B (C26‐bearing mice treated with BCAA+Di‐Ala). The animals underwent a 2‐week pretreatment phase with the supplements (BCAA, alone or in combination with Di‐Ala) dissolved in the drinking water before tumor cell inoculation. The treatment was continued for the duration of the experiment. For the BCAAs, the doses of the three amino acids were as follows: 328 mg/kg of l‐leucine; 164 mg/kg of l‐isoleucine; 164 mg/kg of l‐valine. Di‐Ala was administered at a dose of 328 mg/kg. On Day 12 after C26 implantation, mice were deeply anesthetized, then decapitated, and their tissues quickly collected and weighed.

Concerning the experiments on severe cachexia, the animals were randomized and divided into seven groups: Control (mice without tumor cell inoculation), C26 (C26‐bearing mice treated with vehicle), C26+A (C26‐bearing mice treated with BCAA), C26+B (C26‐bearing mice treated with BCAA+Di‐Ala), C26+OxFu (C26‐bearing mice treated with oxaliplatin/5‐fluorouracil combined chemotherapy), C26+OxFu + A (C26‐bearing mice treated with oxaliplatin/5‐fluorouracil and BCAA), and C26+OxFu + B (C26‐bearing mice treated with oxaliplatin/5‐fluorouracil and BCAA+Di‐Ala). OxFu regimen (50 mg/kg 5‐Fluorouracil and 6 mg/kg oxaliplatin) was administered weekly starting 7 days after C26 inoculation, according to the protocol previously reported [27].

As aforementioned, mice underwent a 2‐week pretreatment phase with the supplements dissolved in the drinking water, at the doses previously employed. The treatment was continued for the duration of the experiment. Body weights were assessed daily and animals that lost 20% of their body weight reached the endpoint and were sacrificed according to the study protocol. Muscle tissues, tumor mass, and plasma samples were collected for subsequent biochemical and morphological analysis, which was performed as previously described [2].

### 2.2 Plasma BCAA Quantification

Estimation of BCAA levels in plasma samples was performed by using a commercial kit (MAK562, Merck Life Science, Milan, Italy) according to the manufacturer's instructions.

### 2.3 Morphological Analysis

Morphological analysis on Tibialis anterior was performed according to the previously described protocol [2, 28]. Briefly, OCT frozen transverse sections were cut by a cryostat (thickness: 10 μm) and collected on Superfrost Plus slides. For each Tibialis anterior, six sections were processed for hematoxylin/eosin staining, dehydrated, and mounted with Eukitt (Kindler GmbH & Co., Germany).

### 2.4 SUnSET Assay

Ex vivo measurement of protein synthesis was assessed by the nonradioactive SUnSET assay, with slight modifications to the previously described protocol [29]. Briefly, muscles were equilibrated for 15 min in oxygenated Krebs–Henseleit solution (119 nM NaCl, 4.7 nM KCl, 2.5 nM CaCl_2_, 1.0 nM MgSO_4_, 25 nM NaHCO_3_, 1.2 nM KH_2_PO_4_, and 1.1 nM glucose), maintained at 37°C. Following equilibration, puromycin was added to the medium at a final concentration of 1 μM. Muscles were then incubated for 1 h, and finally harvested and processed for lysate preparation and Western blot analysis.

### 2.5 Lysate Preparation and Western Blot Analysis

Mouse tissues (TA muscles and C26 tumors) were disrupted in sample buffer (Hepes 10 mM, KCl 10 mM, MgCl_2_ 1.5 mM, NP‐40 0.1%, DTT 0.5 mM, protease, and phosphatase inhibitor cocktail) through 30 s of sonication to produce total lysates. The plasma samples were used as is.

Protein concentrations were measured using Lowry's method, and Laemmli buffer was added to denature cellular proteins. The samples were then boiled at 95°C for 3 min. For electrophoretic separation, 30 μg of protein extract was loaded for TA muscles, and 40 μg was loaded for plasma and tumor samples onto an SDS‐PAGE gel. Proteins were transferred onto nitrocellulose membranes using the Trans‐Blot Turbo system (Bio‐Rad Laboratories, Milan, Italy). The membranes were then blocked with 5% fat‐free milk powder dissolved in DPBS containing 0.1% Tween‐20 (STS0200, Merck Life Science, Milan, Italy) for 1 h at room temperature. Following blocking, the membranes were incubated overnight at 4°C with primary antibodies, as detailed in Table S1. Subsequently, membranes were incubated for 1 h at room temperature with HRP‐conjugated secondary antibodies (Table S1). Protein–antibody immunocomplexes were visualized using Clarity ECL Western blotting reagent (1705061, Bio‐Rad Laboratories, Milan, Italy), and chemiluminescence images were captured using the ChemiDoc MP system (Bio‐Rad Laboratories, Milan, Italy).

Densitometric analysis was conducted using ImageJ software v.154d for Windows 11 (National Institutes of Health, Bethesda, MD, USA). Protein loading was normalized across all samples using Vinculin and GAPDH as housekeeping proteins or Ponceau S staining. The results of the densitometric analysis were expressed in arbitrary units, calculated as the ratio of the target protein band intensity to that of the corresponding housekeeping protein.

### 2.6 RNA Extraction and qRT‐PCR Analysis

Total RNA was extracted from TA muscles using TRI reagent (Merck Life Science, Milan, Italy) according to the manufacturer's protocol. The RNA was treated with DNAse (Ambion, Life Technologies, Italy) to remove genomic DNA and further purified using the RNA Clean‐Up Kit (Zymo, Italy). Subsequently, RNA was reverse transcribed into cDNA using the High‐Capacity cDNA Reverse Transcription Kit (Applied Biosystems) and analyzed by qPCR. Oligonucleotides sequences used for qRT‐PCR are provided in Table S2. Quantitative PCR was conducted in triplicates using SYBR Green IQ reagent (Bio‐Rad Laboratories, Italy) with CFX Connect detection system (Bio‐Rad Laboratories, Italy). Changes in gene expression were represented as “normalized fold induction.” Briefly, *gapdh* was used as a housekeeping gene to normalize the expression levels of each sample; fold change was then calculated by dividing the normalized expression values by the mean expression level of the control group.

### 2.7 ELISA

Plasma interleukin‐6 (IL6) and tumor necrosis factor‐alpha (TNFα) were quantified by using ELISA kits (KMC0061 and BMS607‐3, Thermo Fisher Scientific, Waltham, MA, USA) according to the manufacturer's instructions.

### 2.8 Statistical Analysis

Results presented in this study were expressed as mean ± SD (standard deviation). Statistics were performed by using one‐way analysis of variance (ANOVA), followed by Tukey's *post hoc*. *p* < 0.05 was considered to indicate a statistically significant difference. Statistical analysis and graph editing were carried out using GraphPad Prism 8.4.2 (GraphPad, La Jolla, CA, USA) for Windows 11.

## 3 Results

### 3.1 BCAA Formulations, Alone or in Combination With Di‐Ala, Protect C26 Tumor‐Bearing Mice From Weight Loss and Muscle Wasting

We first investigated whether the formulations containing BCAA (called formulation “A”) or BCAA with Di‐Ala peptide (called formulation “B”) could attenuate weight loss in C26 tumor‐bearing mice.

**FIGURE 1:**
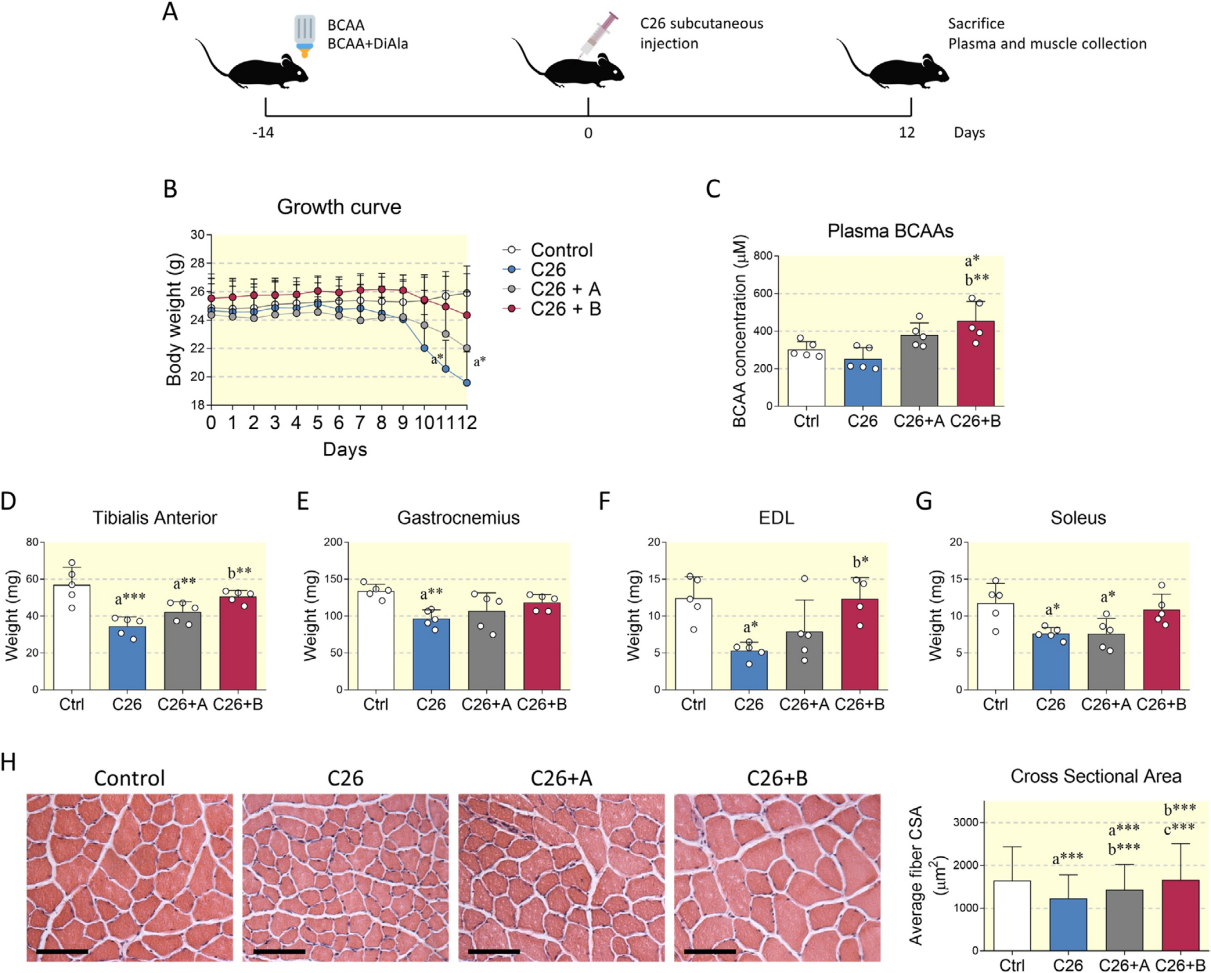
Effect of BCAA formulations on body weight and muscle loss. (A) Schematic representation of the experimental design. BCAA formulations were administered throughout the experimental period. (B) Body weight chart of control mice, C26 tumor‐bearing mice (C26), C26 tumor‐bearing mice treated with BCAA (C26+A) and C26 tumor‐bearing mice treated with BCAA+Di‐Ala (C26+B). *N* = 5 individuals for each experimental group. (C) Plasma concentration of BCAA levels in the above experimental groups. (D–G) Tibialis anterior, Gastrocnemius, EDL, and soleus were weighted 12 days after C26 cell implantation. *N* = 5. (H) Representative images of hematoxylin/eosin staining performed on Tibialis anterior and relative calculation of mean cross‐sectional area (CSA). For CSA determination, 1000–1300 fibers (~400 fibers per animal) were analyzed for each experimental condition. *N* = 3 independent experiments. Scale bar: 100 μm. Data are represented as means ± SD. Statistical analysis was performed by using one‐way ANOVA followed by Tukey's post hoc test. “a” indicates statistical significance versus Ctrl group; “b” indicates statistical significance versus C26 group. *p < 0.05, **p < 0.01, ***p < 0.001.

Animals were pretreated with the BCAA formulations for 2 weeks, and the body weight of C26 tumor‐bearing mice was therefore monitored daily from the time of inoculation with adenocarcinoma cells (Figure 1A). Consistent with previous observations [2], tumor‐bearing mice (C26 group) progressively lost body weight starting 10 days after the inoculation of C26 cells, reaching statistical significance at 11 and 12 days. In contrast, C26‐bearing mice treated with BCAA (C26+A group) exhibited protection from weight loss, an effect that was even more pronounced in animals treated with BCAA + Di‐Ala (C26+B group) (Figure 1B).

Twelve days after cell inoculation, mice from all the experimental groups were sacrificed and plasma and hindlimb muscles were collected to assess BCAA levels and muscle wasting, respectively. Cachectic mice exhibited a slight, yet nonsignificant, reduction in the concentration of plasma BCAA. Notably, treatment with both nutraceutical formulations resulted in an upward trend in BCAA levels, with the co‐administration of Di‐Ala inducing a more marked and statistically significant increase (Figure 1C). Cancer‐induced cachexia significantly promoted muscle loss in the Tibialis anterior (Figure 1D), Gastrocnemius (Figure 1E), Extensor digitorum longus (EDL) (Figure 1F) and Soleus (Figure 1G). Muscle wasting was reduced in the Tibialis anterior, EDL, and Gastrocnemius after BCAA administration, with the exception of the Soleus. Intriguingly, Di‐Ala boosted the protective effects induced by BCAA in all muscles considered in this study, including the Soleus.

The effects on muscle weight were further confirmed by hematoxylin/eosin staining of frozen sections of the Tibialis anterior. Notably, cross‐sectional area (CSA) was significantly reduced in C26‐bearing mice. However, BCAA administration effectively counteracted the reduction in fiber diameter, with Di‐Ala supplementation having an enhancing effect (Figure 1H, Figure S1A).

### 3.2 BCAA Formulations, Alone or in Combination With Di‐Ala, Prevent the Induction of Catabolic Pathways in the Atrophying Muscle

**FIGURE 2:**
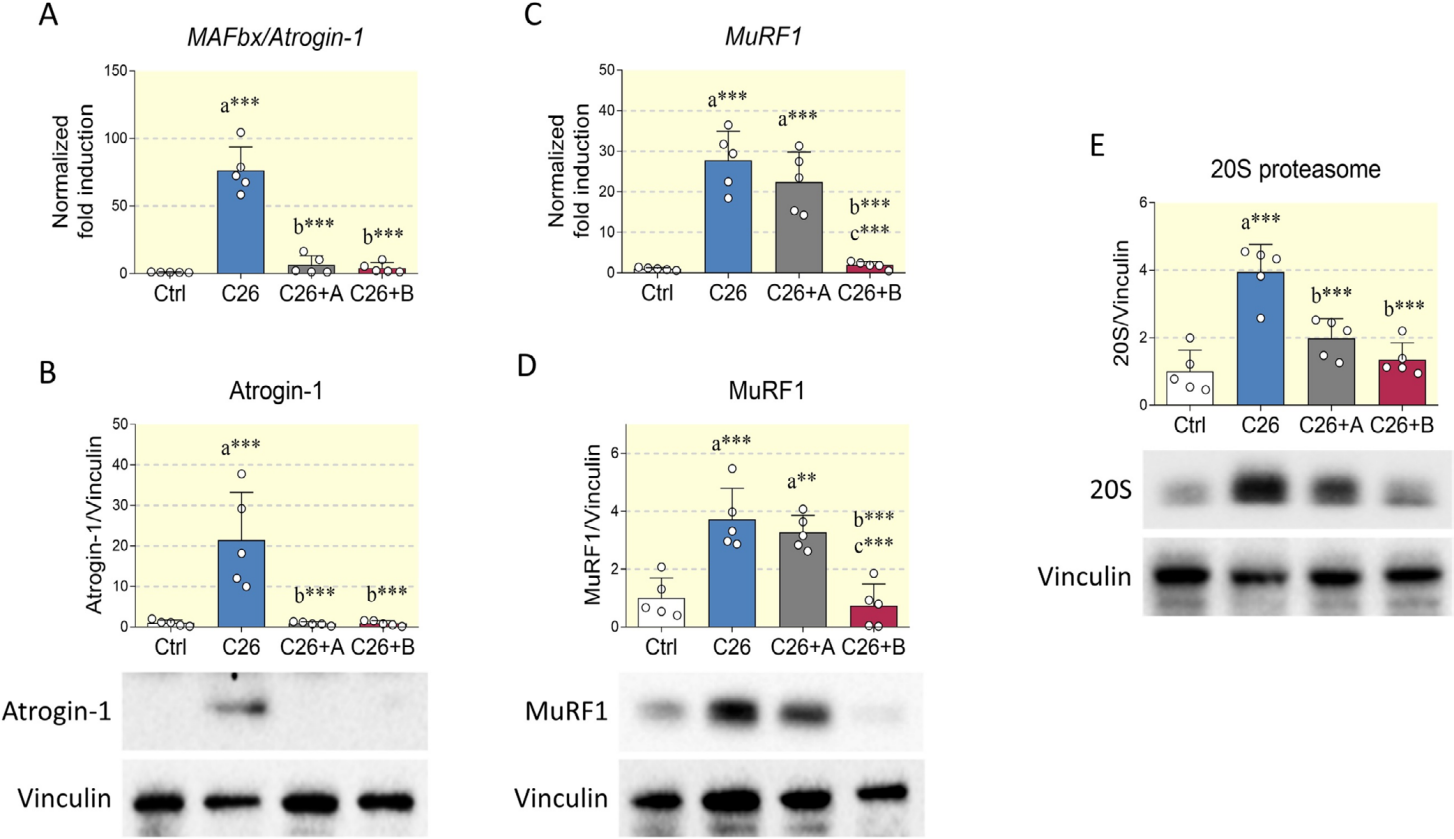
Effects of amino acid supplementation on the expression of atrogenes in the Tibialis anterior. (A) Total RNA was extracted from control mice (Ctrl), C26 tumor‐bearing mice (C26), C26 tumor‐bearing mice treated with BCAA (C26+A) and C26 tumor‐bearing mice treated with BCAA+Di‐Ala (C26+B), and expression levels of Atrogin‐1 were measured by quantitative RT‐PCR. *N* = 5 animals per group. (B) Representative Western blot and densitometric analysis of Atrogin‐1 in the experimental groups reported in (A). Vinculin was chosen as housekeeping protein. *N* = 5 mice. (C) Transcript levels of MuRF1 of experimental groups previously described. *N* = 5 animals. (D, E) Representative Western blot and densitometric analysis of MuRF1 and 20S proteasome in the experimental groups reported above. Vinculin served as loading control. *N* = 5 animals per group. Data are represented as means ± SD. Statistical analysis was performed by using one‐way ANOVA followed by Tukey's post hoc test. “a” indicates statistical significance versus Ctrl group; “b” indicates statistical significance versus C26 group. **p < 0.01, ***p < 0.001.

Muscle wasting during cancer cachexia is induced by the upregulation of the UPS. Specifically, protein catabolism is associated with the build‐up of muscle‐specific E3 ubiquitin ligases such as Atrogin‐1 and muscle RING‐finger protein‐1 (MuRF1) [1]. The results obtained in this study support this evidence, as muscles from C26‐bearing mice were associated with a dramatic increase in Atrogin‐1 at both mRNA and protein levels. Interestingly, both BCAA and BCAA+Di‐Ala administration normalized the expression of this catabolic enzyme (Figure 2A,B). A similar trend was observed for MuRF1 transcript and protein levels. However, only BCAA+Di‐Ala was able to properly restore its basal express (Figure 2C,D). The increased expression of muscle ubiquitin ligases was also accompanied by a sustained protein expression of the 20S proteasome, which was suppressed by the administration of BCAA and BCAA+Di‐Ala formulations (Figure 2E).

**FIGURE 3:**
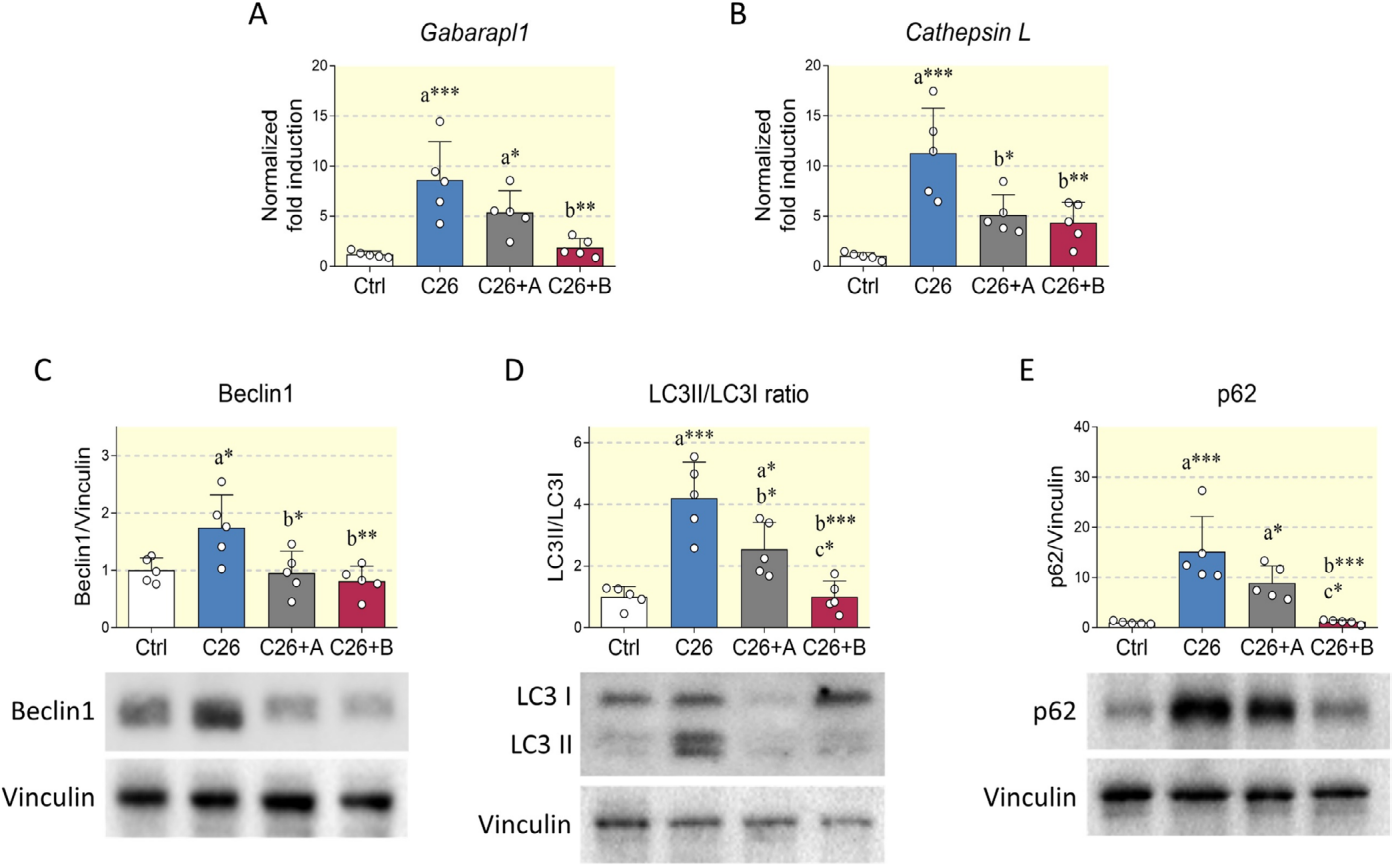
Effects of amino acid supplementation on the expression of autophagolysosomal markers in the Tibialis anterior. (A, B) Total RNA was extracted from control mice (Ctrl), C26 tumor‐bearing mice (C26), C26 tumor‐bearing mice treated with BCAA (C26+A) and C26 tumor‐bearing mice treated with BCAA+Di‐Ala (C26+B), and expression levels of Gabarapl1 and CathepsinL were measured by quantitative RT‐PCR. *N* = 5 animals per group. (C–E) Representative Western blots and densitometric analysis of Beclin1, LC3, and p62 in the experimental groups described above. Vinculin was chosen as loading control. *N* = 5 mice. Data are represented as means ± SD. Statistical analysis was performed by using one‐way ANOVA followed by Tukey's post hoc test. “a” indicates statistical significance versus Ctrl group; “b” indicates statistical significance versus C26 group. *p < 0.05, **p < 0.01, ***p < 0.001.

Several reports have highlighted that cancer cachexia leads to muscle loss not only through proteasomal degradation, but also through activation of autophagic degradation [2]. Therefore, we estimated the main effectors involved in the autophagolysosomal pathway. *Gabarapl1* mRNA expression was significantly upregulated in muscles from C26‐bearing mice, which was attenuated only by BCAA+Di‐Ala (Figure 3A). Similarly, *CathepsinL* transcript was also increased in experimental cancer cachexia. In this case, both the formulations reduced the expression of this lysosomal protease (Figure 3B). Western blot analysis revealed that cancer cachexia promoted an increase in the expression of Beclin1, which is involved in the initiation of autophagy, whereas both BCAA and BCAA+Di‐Ala prevented its increase (Figure 3C). The LC3II/LC3I ratio, a common method to assess autophagy activation, is increased in C26‐bearing mice. In contrast, BCAA administration reduced the LC3II/LC3I ratio and this effect was further enhanced upon coadministration of Di‐Ala (Figure 3D). Similar evidence was obtained by analyzing the expression of p62 (Figure 3E), an autophagy marker frequently upregulated in cachexia‐induced muscle atrophy [30]. Taken together, these results indicate that autophagic degradation is upregulated in cachectic muscles and is attenuated by BCAA, with Di‐Ala having a boosting effect.

### 3.3 Effects of BCAA, Alone or in Combination With Di‐Ala, on Signaling Pathways Regulating Muscle Mass

**FIGURE 4:**
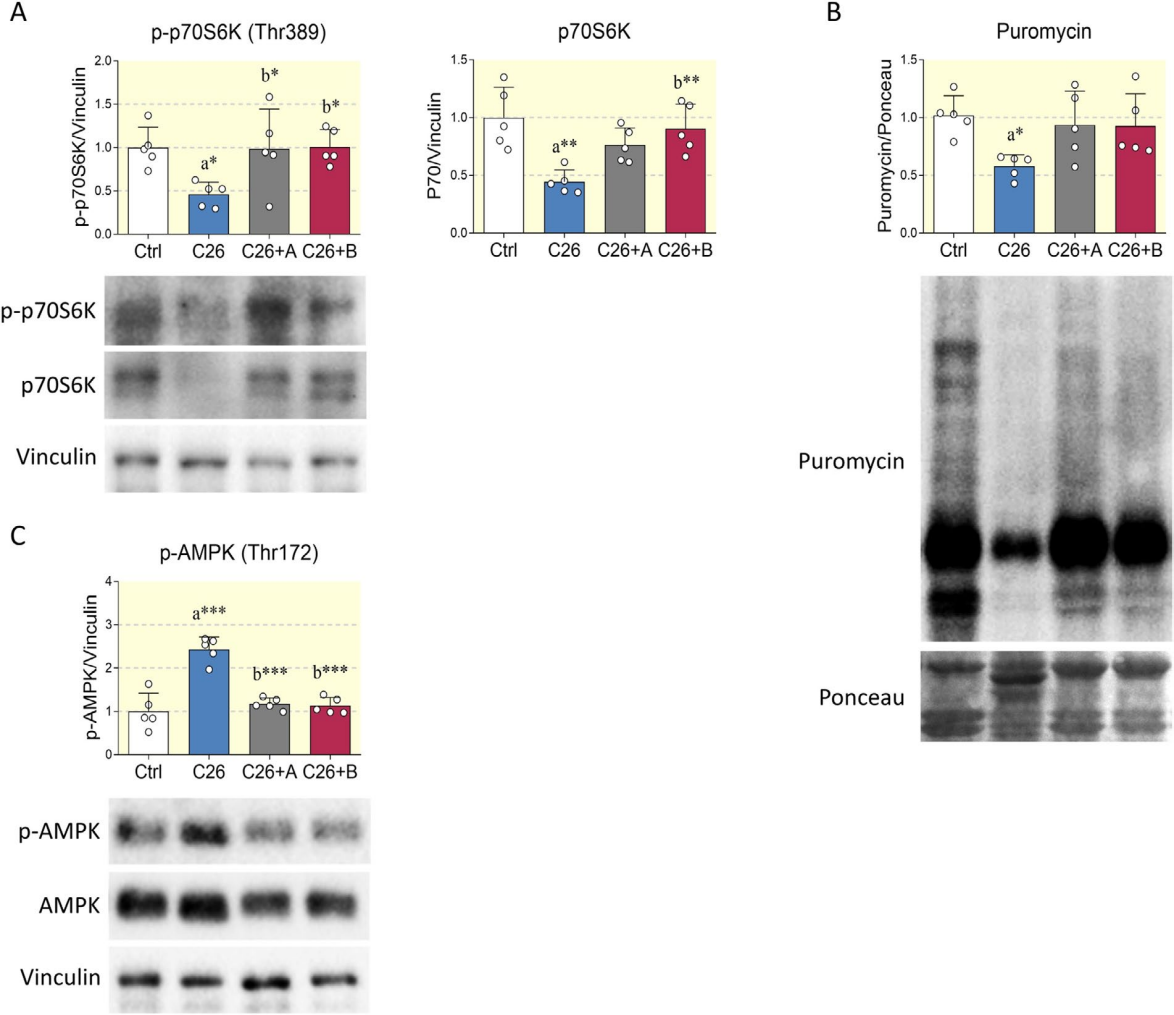
Effects of BCAA formulations on p70S6K and AMPK activation. (A) Representative Western blots and densitometric analysis of phopsho‐p70S6K in the Tibialis anterior of control mice (Ctrl), C26 tumor‐bearing mice (C26), C26 tumor‐bearing mice treated with BCAA (C26+A) and C26 tumor‐bearing mice treated with BCAA+Di‐Ala (C26+B). Vinculin was chosen as loading control. *N* = 5 mice. (B) Protein synthesis was estimated by assessing puromycin incorporation into proteins from Tibialis anterior of mice treated as in (A). *N* = 5 animals. (C) Representative Western blot and densitometric calculation of phospho‐AMPK in Tibialis anterior muscles. Vinculin served as housekeeping protein. *N* = 5 mice per group. Data are represented as means ± SD. Statistical analysis was performed by using one‐way ANOVA followed by Tukey's post hoc test. “a” indicates statistical significance versus Ctrl group; “b” indicates statistical significance versus C26 group. *p < 0.05, **p < 0.01, ***p < 0.001.

The Akt/mTOR/p70S6K pathway is required for skeletal muscle hypertrophy as it mediates the induction of protein synthesis and the concomitant reduction in atrogenes expression and autophagy activation [31]. In this context, several studies suggest that muscle atrophy during cachexia is mediated by the suppression of the Akt/mTOR/p70S6K axis. The results collected in this study support this notion, as p70S6K activating phosphorylation and total protein expression are significantly reduced in cachectic muscles. Interestingly, both BCAA and BCAA+Di‐Ala prevented the changes observed in p70S6K, suggesting recovery in protein synthesis (Figure 4A). To ascertain this hypothesis, we performed the SUnSET assay to assess protein synthesis through the incorporation of puromycin into nascent proteins. In line with the observed p70S6K activation state, cancer cachexia markedly impaired the rate of protein translation, an effect that was significantly counteracted by the two nutraceutical formulations (Figure 4B). However, the coadministration of Di‐Ala did not confer any further benefit beyond that achieved by BCAA alone. In addition to the dysregulation of the Akt/mTOR/p70S6K pathway, muscle atrophy induced by cancer cachexia is also driven by the hyperactivation of AMP‐activated protein kinase (AMPK), which not only suppresses the mTOR/p70S6K axis, but also activates autophagy and upregulates the transcription of genes involved in muscle protein catabolism via autophagolysosomal and proteasomal systems [2, 32, 33, 34]. In our experimental model, both BCAA and BCAA+Di‐Ala supplementations abolished AMPK hyperphosphorylation induced by C26 tumor cell inoculation (Figure 4C). Collectively, these results suggest that BCAA administration, alone or in combination with Di‐Ala, prevents the suppression of anabolic pathways as well as the activation of catabolic signaling cascades occurring in cachexia‐mediated muscle atrophy.

**FIGURE 5:**
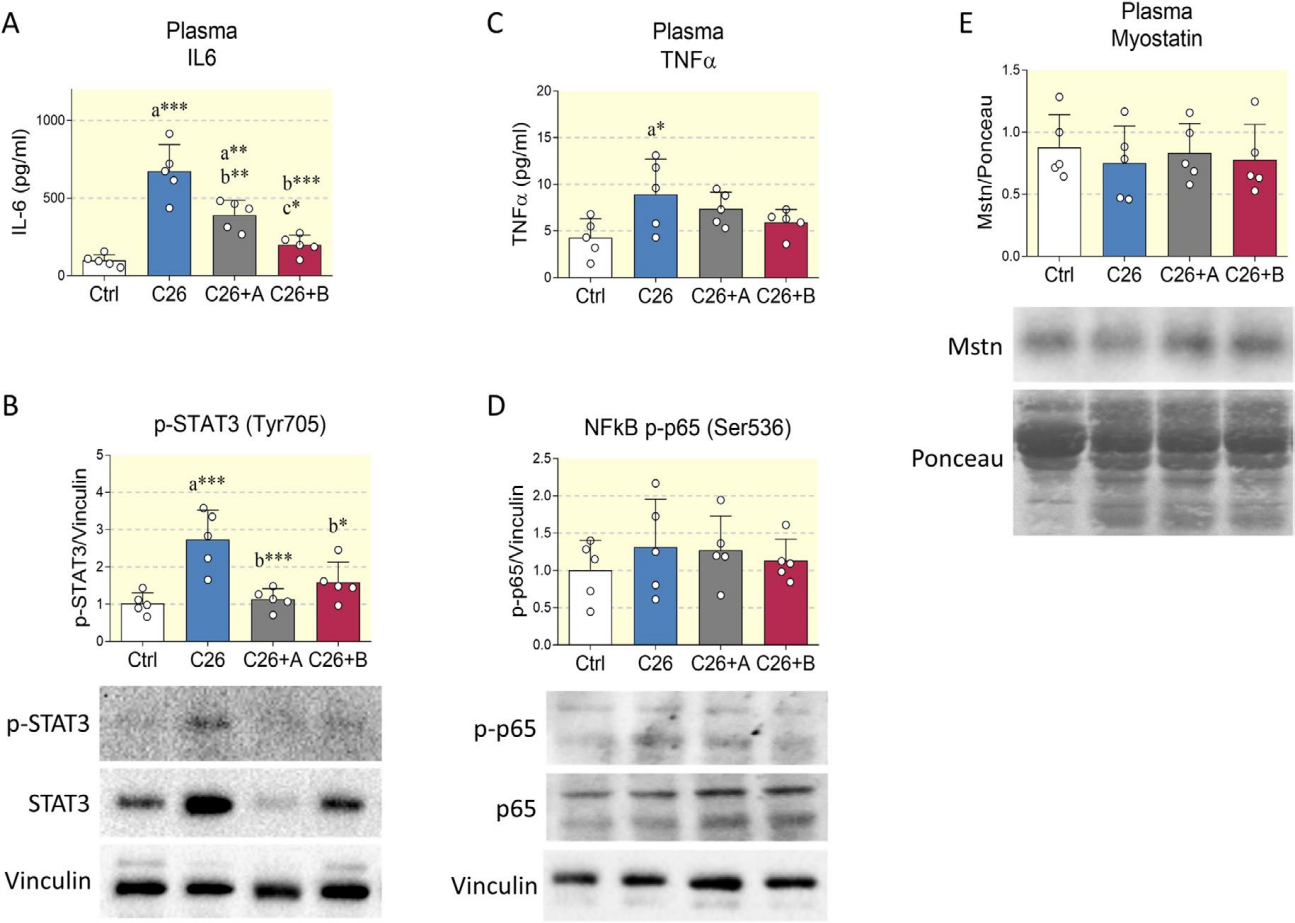
Effect of BCAA supplementation on procachectic pathways. (A) ELISA on plasma IL6 from control mice (Ctrl), C26 tumor‐bearing mice (C26), C26 tumor‐bearing mice treated with BCAA (C26+A), and C26 tumor‐bearing mice treated with BCAA+Di‐Ala (C26+B). *N* = 5 mice. (B) Representative Western blot and densitometric analysis of phopsho‐STAT3 in the Tibialis anterior of the experimental groups reported as in (A). Vinculin was chosen as loading control. *N* = 5 mice. (C) ELISA on plasma TNFα from mice treated as previously described. *N* = 5 animals per group. (D, E) Representative Western blots and densitometric analysis of phospho‐p65 (NF‐kB) and myostatin in the Tibialis anterior and plasma, respectively. Vinculin or Ponceau S staining served to normalize protein loading. *N* = 5 mice. Data are represented as means ± SD. Statistical analysis was performed by using one‐way ANOVA followed by Tukey's post hoc test. “a” indicates statistical significance versus Ctrl group; “b” indicates statistical significance versus C26 group. *p < 0.05, **p < 0.01, ***p < 0.001.

A growing body of evidence suggests a pivotal role for circulating procachectic molecules secreted by the tumor and by other tissues of the host. Notably, several humoral factors, such as IL6, TNFα, and myostatin, drive muscle atrophy through the activation of catabolic signaling pathways [2]. As previously reported [35], we observed heightened plasma IL6 levels in cachectic mice, as evidenced by Western blot and ELISA assay. Surprisingly, we found that the accumulation of plasma IL6 was significantly mitigated by BCAA, with an even more pronounced effect in the presence of Di‐Ala supplementation (Figure 5A, Figure S1B). Furthermore, the activating phosphorylation of Signal Transducer and Activator of Transcription 3 (STAT3) was confirmed as a marker of skeletal muscle responsiveness to IL6 (Figure 5B). In addition, no changes in IL6 expression were observed in lysates from Tibialis anterior at both mRNA and protein level (Figure S1C,D), indicating that both cancer cachexia and BCAA supplementation impact circulating IL6 levels without affecting local IL6 production in skeletal muscle.

Subsequently, the effect of BCAA supplementation on TNFα was assessed. According to previous reports, circulating TNFα levels were increased in cachectic mice [36], and tended to decrease upon administration of both BCAA and the BCAA+Di‐Ala combination (Figure 5C). Consequently, the activation state of nuclear factor kappa B (NF‐kB) p65, a pivotal target of TNFα signaling, was evaluated in skeletal muscle. Despite the changes in plasma TNFα, no alterations were observed in the activating phosphorylation of NF‐kB p65 in the Tibialis anterior (Figure 5D). Myostatin, a negative regulator of muscle growth, has been shown to be frequently involved in the induction of muscle atrophy [37]. The expression of this growth factor remained unaltered in both the plasma and the muscle (Figure 5E, Figure S1E). Taken together, these results suggest that, according to previous findings [2], IL6 is the main driver of muscle atrophy in the experimental model of C26‐induced cachexia, whereas TNFα and myostatin only have marginal roles.

### 3.4 Impact of BCAA Formulations on Severe Cachexia, in Combination With Standard Chemotherapy

Several studies have shown that nutritional approaches based on protein supplementation could delay the onset of severe cachexia, thereby improving patients' response to cancer therapy, quality of life, and survival [38, 39].

**FIGURE 6:**
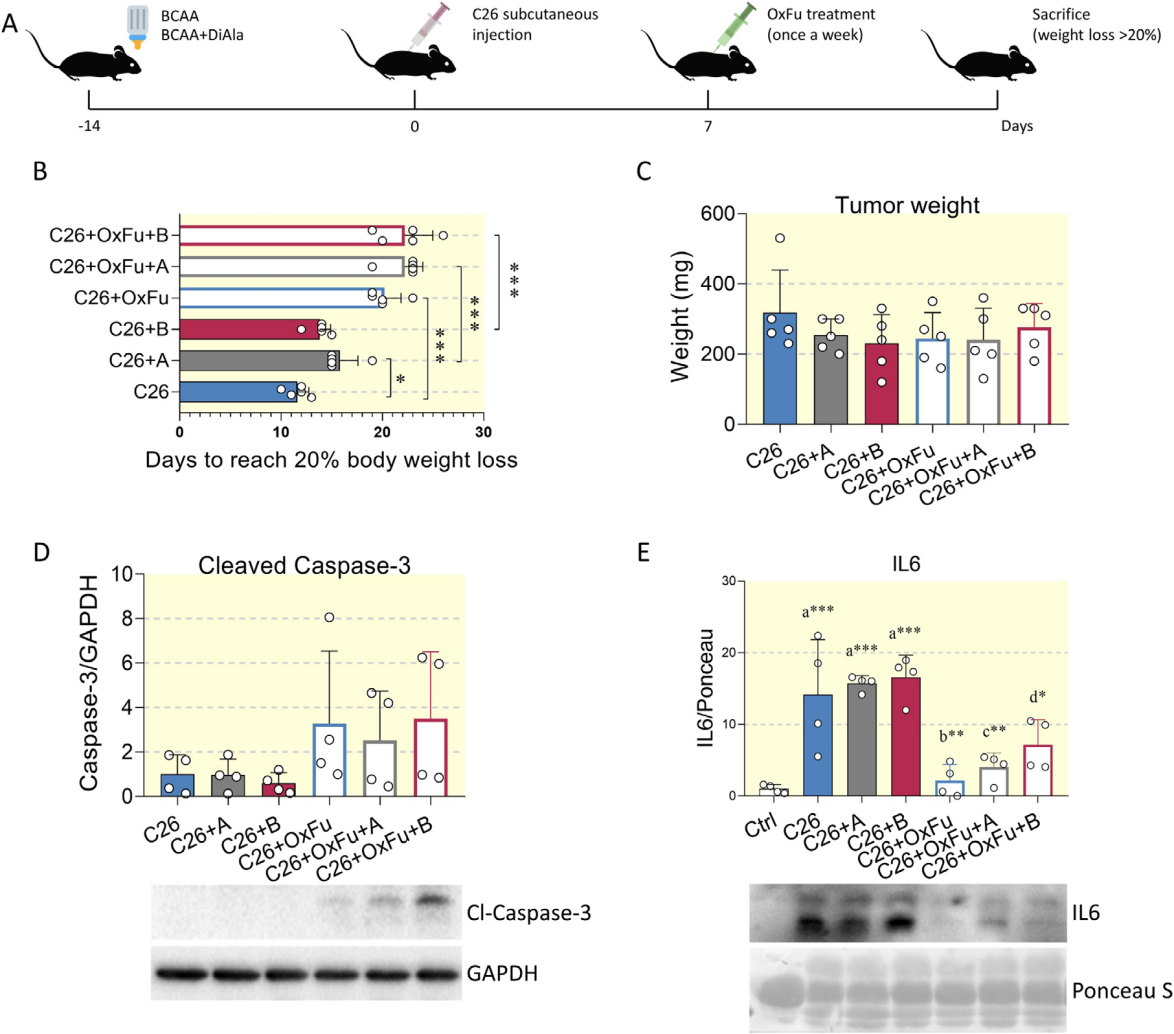
Effects of chemotherapy and BCAA formulations on severe cachexia and tumor growth. (A) Illustration of the experimental design. BCAA supplements were administered throughout the experimental period. Animals were sacrificed when they reached a stage of severe cachexia (body weight loss > 20%. (B) Chart showing time to reach severe cachexia (body weight loss > 20%) in C26 tumor‐bearing mice (C26), C26 tumor‐bearing mice treated with BCAA (C26+A), and C26 tumor‐bearing mice treated with BCAA+Di‐Ala (C26+B). Three other groups received OxFu regimen (50 mg/kg 5‐Fluorouracil and 6 mg/kg oxaliplatin, once a week per i.p.) as chemotherapy (C26+OxFu, C26+OxFu+A, C26+OxFu+B). *N* = 5 mice. (C) Tumor weight from animals treated as in (A). *N* = 5 animals. (D) Representative Western blot and densitometric analysis of cleaved caspase‐3 in C26 tumors of the experimental groups reported as in (B). GAPDH was chosen as loading control. *N* = 4 mice. (E) Representative Western blot and densitometric analysis of plasma IL6 from Ctrl, C26, C26+A, C26+B, C26+OxFu, C26+OxFu+A, and C26+OxFu+B experimental groups. Ponceau S staining served as loading control. *N* = 4 animals. Data are represented as means ± SD. Statistical analysis was performed by using one‐way ANOVA followed by Tukey's post hoc test. “a” indicates statistical significance versus Ctrl group; “b” indicates statistical significance versus C26 group. *p < 0.05, **p < 0.01, ***p < 0.001.

Therefore, we decided to extend our data by evaluating the role of BCAA administration, alone or in combination with Di‐Ala, in the progression to a stage of severe cachexia (i.e., a weight loss of more than 20%), in the presence or absence of standard chemotherapy for colorectal cancer (combined administration of oxaliplatin and 5‐fluorouracil) (Figure 6A). Consistent with previous reports showing an increase in life expectancy [40, 41], chemotherapy decelerated the progression to end‐stage disease (Figure S2A). Notably, chemotherapy effectively delays the onset of severe cancer cachexia (C26+OxFu: 20.2 days) when compared to nontreated animals (C26: 11.6 days). Although statistical significance was not reached in most cases, the administration of BCAA and BCAA+Di‐Ala formulations, alone or in combination with a chemotherapy regimen, slightly slowed the occurrence of severe cachexia (C26+A: 15.8 days; C26+B: 13.8 days; C26+OxFu+A: 22.2 days; C26+OxFu+B: 22.2 days) (Figure 6B). It is noteworthy that the addition of Di‐Ala did not result in a demonstrable advantage in the mitigation of severe cachexia when compared to BCAA alone.

Amino acids have been demonstrated to play a pivotal role in facilitating tumor development and growth [42]. To exclude the possibility that BCAA supplementation could positively affect the metabolism of cancer cells, the tumor mass was evaluated when the animals reached a condition of severe cachexia. The assessment of tumor weight demonstrated that BCAA supplementation did not exert a significant influence on the growth of the C26 tumor mass (Figure 6C). The growth and survival of cancer cells is frequently driven by the hyperactivation of kinases, such as ERK1/2 and Akt, which are involved in the regulation of cell metabolism and proliferation [43]. The lack of any change in tumor growth is consistent with the activating phosphorylation states of ERK1/2 and Akt, which remained unaffected among the experimental conditions evaluated in the study (Figure S2B,C). In addition, the cleavage of caspase‐3, a well‐established apoptosis marker utilized in both preclinical and clinical analyses, was examined. The administration of BCAA and BCAA+Di‐Ala did not result in any alterations in the tumor expression levels of cleaved caspase‐3 when compared to nontreated cachectic animals. However, chemotherapy treatment led to an increase in cleaved caspase‐3, although the substantial intra‐group variability precluded the attainment of statistical significance (Figure 6D). The data suggest that chemotherapy regimens exert cytotoxic effects, leading to the induction of apoptotic pathways. However, since tumor growth was comparable among the diverse experimental groups (Figure 6C), it can be suggested that tumor cells, over time, develop drug resistance through compensatory mechanisms which counteract the antineoplastic activity of chemotherapeutic agents. As shown above, both BCAA and BCAA+Di‐Ala significantly reduced the elevation of plasma IL6 in moderate cachexia. We therefore investigated whether this effect was maintained in the context of severe cachexia. Neither BCAA nor BCAA+Di‐Ala mixtures were able to prevent IL6 elevation during severe cachexia. Conversely, chemotherapy administration significantly attenuated IL6 expression (Figure 6E). However, this event was not sufficient to prevent the development of severe cachexia, suggesting that other mechanisms compensate for the suppression of IL6 induced by antineoplastic agents to promote a severe cachectic condition.

## 4 Discussion

Cancer cachexia‐induced muscle atrophy represents a significant clinical challenge that currently lacks effective therapeutic interventions. Its management is of critical importance, as it not only severely impacts patients' mobility, but also reduces the efficacy of chemotherapeutic approaches, thereby negatively affecting prognosis [44, 45, 46].

In this work, we explored the ability of BCAA supplementation, alone or in combination with the dipeptide Di‐Ala, to counteract muscle wasting during cancer cachexia.

BCAA supplementation reduced body weight loss and muscle atrophy in the Tibialis anterior, EDL, and Gastrocnemius. Notably, the addition of Di‐Ala to the BCAA mixture further enhanced these effects. It is interesting to note that in the Soleus, which is mainly composed of slow‐twitch fibers, muscle wasting was only prevented when BCAA were coadministered in the presence of Di‐Ala.

The different response of slow‐twitch and fast‐twitch dominant muscles to BCAA has been reported previously and may be related to specific differences in BCAA catabolism [47]. At the molecular level, we revealed that Di‐Ala increases protection against muscle atrophy because this dipeptide potentiates the inhibitory effects of BCAA on the expression of certain catabolic genes and proteins belonging to the UPS or autophagy‐lysosomal system, such as MuRF1, Gabarapl1, LC3, and p62.

p70S6K and AMPK play crucial opposing roles in the regulation of muscle mass, as the mTOR/p70S6K axis promotes protein biosynthesis [48], whereas AMPK induces protein catabolism [2]. Our results show that both BCAA and BCAA+Di‐Ala supplementation effectively restored the diminished activation of p70S6K and rescued the associated decline in protein synthesis. Furthermore, these nutritional interventions normalized the exaggerated activating phosphorylation of AMPK. This is in line with previous findings showing that BCAA are potent stimulators of the mTOR/p70S6K pathway [49, 50]. BCAA not only serve for protein biosynthesis, but their catabolism can also be used to produce ATP through the generation of acetyl‐CoA or succinyl‐CoA, which enter the Krebs cycle [51]. It can therefore be speculated that by increasing the ATP/AMP ratio, BCAA‐based dietary supplements may directly contribute to the reduction in AMPK activity observed in this study. Furthermore, the occurrence of an interplay between AMPK and p70S6K, already demonstrated in other physiopathological contexts [33, 52], may be involved in determining the changes observed here. Collectively, these results suggest that Di‐Ala supplementation does not amplify the anabolic effects of BCAA on protein synthesis but rather augments their capacity to suppress catabolic pathways.

The activation of pathways controlling muscle mass during cachexia is influenced not only by the intracellular metabolic state, but also by a variety of extracellular signals, including growth factors and cytokines. In this context, increasing evidence highlights that IL6 has a central role in AMPK activation in both healthy and atrophying muscle [2, 34, 53]. We unexpectedly found that administration of BCAA led to a significant reduction in the plasma levels of IL6 in moderate cachexia. This effect was further enhanced when BCAA was combined with Di‐Ala. To our knowledge, this is the first report showing the impact of BCAA, alone and in combination with Di‐Ala, on systemic IL6 levels. Given the multifaceted role of circulating IL6 in the cachectic syndrome, this phenomenon suggests that the beneficial effects of these nutraceutical formulations are not limited to the prevention of muscle wasting but may be related to other pathological features of cachexia, which warrant further study. On the contrary, both the formulations tested in this work did not affect local IL6 produced by the muscle. This divergent effect on local versus systemic IL6 sustains the advantageous role of the nutraceutical supplementations evaluated in this study, considering that muscle‐produced IL6 exerts beneficial autocrine and paracrine effects on muscle energy metabolism. Conversely, chronic inflammation associated with cancer cachexia leads to plasma IL6 elevation, which promotes muscle atrophy [54].

We also investigated whether BCAA and BCAA+Di‐Ala mixtures could slow the progression through a stage of severe cachexia, even in the presence of standard chemotherapy. As demonstrated in earlier reports [40, 41], chemotherapy provides an unquestionable benefit in delaying the development of severe cachexia. Nonetheless, in accordance with extant data, while chemotherapy did not negatively impact overall body mass, subsequent research should specifically focus on skeletal muscle weight, as chemotherapeutic agents may cause a slight but significant worsening of muscle atrophy when given during cancer cachexia [55]. Despite no statistical significance, both BCAA and BCAA+Di‐Ala showed a tendency to further retard the onset of severe cachexia, even in the presence of chemotherapy. The modest effect on delaying severe cachexia, as well as the absence of the boosting effect induced by Di‐Ala, suggests that BCAA‐based supplements are more effective in attenuating cachexia‐induced dyshomeostasis during the early stages of the disease, when the body loss is mild or moderate. Accordingly, both formulations failed to prevent the increase in plasma IL‐6 levels during severe cachexia. This is likely since the anti‐inflammatory properties of BCAAs, previously demonstrated in various pathological conditions such as catabolic states, cancer, burns, and sepsis [56], may not be sufficient to contrast the progressive and persistent chronic inflammation occurring during the advanced stages of cachexia.

C26 tumor‐bearing mouse is a well‐established and advantageous model for studying cancer cachexia, as body and tissue depletion occurs in the presence of small tumor burden, similarly to human cancer. However, a major limitation lies in the rapid progression of body consumption, which may restrict the therapeutic window [57]. In this context, the scarce efficacy of BCAA formulations observed in the stage of severe cachexia may be attributable to the accelerated course of the disease in this model. Thus, to exclude model‐specific constraints and better evaluate the therapeutic potential of these interventions in late stages of cancer cachexia, future investigations should employ alternative models characterized by a more gradual disease progression, which more closely mirrors the clinical trajectory observed in humans.

Taken together, the results collected in this study support the notion that BCAA‐based supplements may be useful in the management of muscle atrophy associated with mild‐to‐moderate cancer cachexia, underlying the relevance of innovative formulations containing Di‐Ala, capable of increasing BCAA bioavailability. This is in line with previous reports, showing that Di‐Ala supplementation to BCAA mixtures is more effective in preventing muscle atrophy in diverse physiopathological conditions, such as age‐related sarcopenia and muscle disuse [22, 23]. At the same time, this exploratory project laid the groundwork for future preclinical and clinical studies aimed at evaluating the impact of BCAA and Di‐Ala formulations on cachexia‐related conditions other than muscle atrophy, including adipose tissue wasting, lipid dyshomeostasis, liver alterations, and appetite loss, as all these physiopathological aspects may be influenced by nutritional supplements [58, 59, 60]. Differently from BCAA, Di‐Ala is not currently employed in clinical practice as a nutraceutical component, and existing evidence is primarily limited to preclinical studies demonstrating its safety and efficacy in murine models of disuse‐ and age‐related muscle atrophy [22, 23]. Nonetheless, its potential for clinical translation is particularly promising, as Di‐Ala is a naturally occurring dipeptide produced during protein digestion and physiologically absorbed by the human intestinal epithelium via peptide transporters, including PEPT1 [26, 61, 62].

## Author Contributions

**Mayra Colardo:** data curation, formal analysis, investigation, methodology, validation, visualization, writing – original draft. **Noemi Martella:** formal analysis, investigation, methodology, validation, writing – review and editing. **Michela Varone:** investigation, writing – review and editing. **Daniele Pensabene:** investigation, writing – review and editing. **Giuseppina Caretti:** funding acquisition, methodology, resources, writing – review and editing. **Gianluca Bianchini:** conceptualization, funding acquisition, resources, writing – review and editing. **Andrea Aramini:** conceptualization, funding acquisition, resources, writing – review and editing. **Marco Segatto:** conceptualization, data curation, formal analysis, funding acquisition, investigation, methodology, project administration, software, resources, supervision, validation, visualization, writing – original draft.

## Conflicts of Interest

Authors A.A. and G.B. were employed by Dompé farmaceutici S.p.A. The remaining authors declare that the research was conducted in the absence of any commercial or financial relationships that could be construed as a potential conflicts of interest.

## Supporting information


Data S1.

## Data Availability

The data that support the findings of this study are available on request from the corresponding author. The data are not publicly available due to privacy or ethical restrictions.
