# Supplementary material for: Branched‐Chain Amino Acids and Di‐Alanine Supplementation Attenuates Muscle Atrophy in a Murine Model of Cancer Cachexia
Source: Acta Physiol (Oxf). 2025 May 31;241(7):e70067. doi: 10.1111/apha.70067 (PMC12125566; doi:10.1111/apha.70067)
Supplement: Supplementary file 1 — Data S1. [file APHA-241-e70067-s001.pdf]

Supplementary Material

Supplementary Figure 1

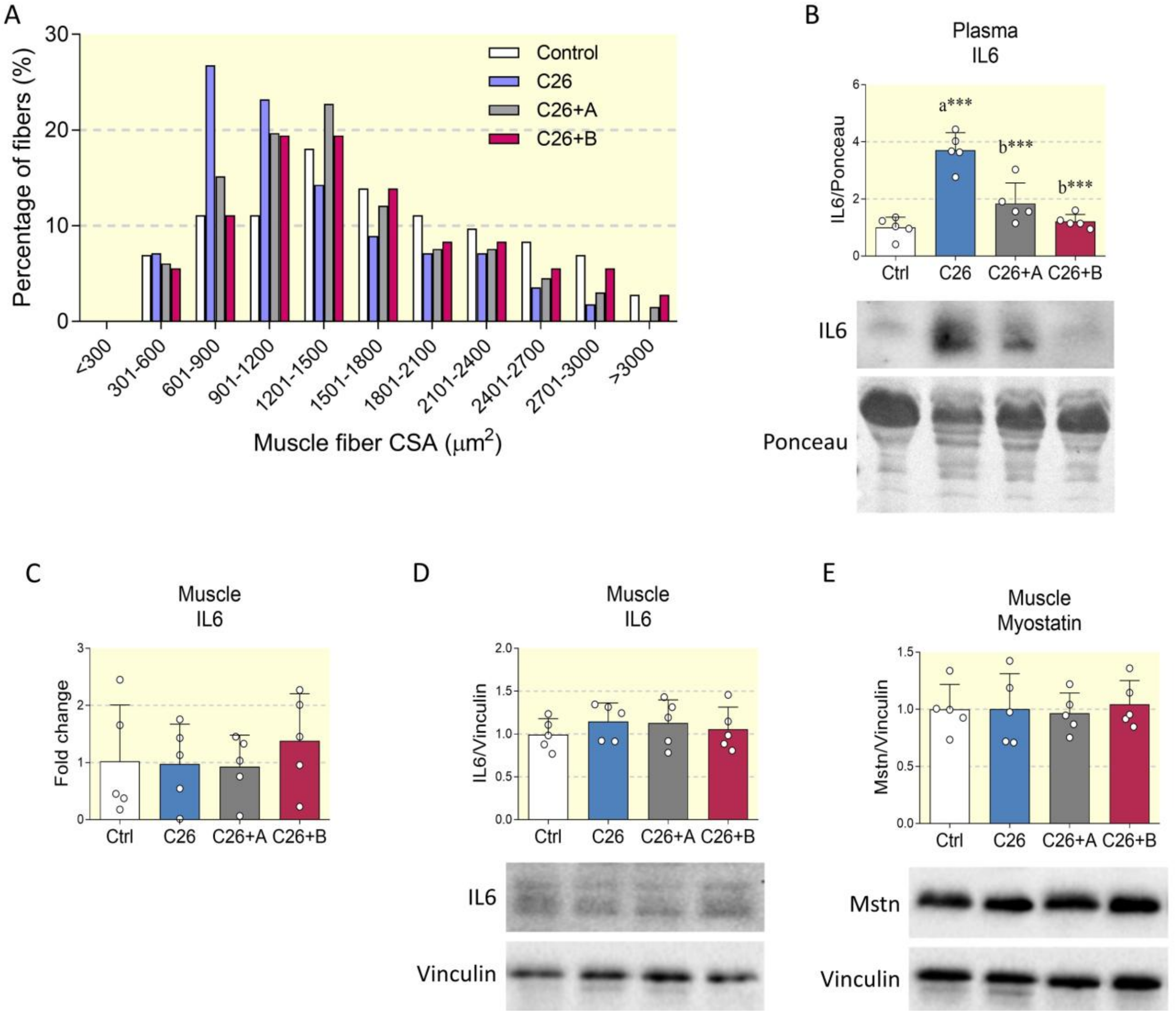

Figure S1. Frequency histogram of muscle CSA and expression of IL6 and myostatin. (A) Calculation of cross sectional area (CSA) in the Tibialis anterior from control mice (Ctrl), C26-tumor bearing mice (C26), C26-tumor bearing mice treated with BCAA (C26+A) and C26-tumor bearing mice treated with BCAA+Di-Ala (C26+B). N=3 independent experiments. (B) Representative Western blot and densitometric analysis of plasma IL6 from animals treated as in (A). Ponceau S staining was chosen as loading control. N=5 mice. (C) Total RNA was extracted from Tibialis anterior and IL6 mRNA was analyzed by qRT-PCR. N=5 animals for each experimental group as previously reported. (D-E) Representative Western blots and densitometric analysis of IL6 and myostatin in the

experimental groups described above. Vinculin was chosen as housekeeping protein to normalize protein loading. N=5 mice. Data are expressed as means±SD. Statistical analysis was performed by using one-way ANOVA followed by Tukey's post hoc test. "a" indicates statistical significance vs Ctrl group; "b" indicates statistical significance vs C26 group. *** $p < 0.001$.

Supplementary Figure 2

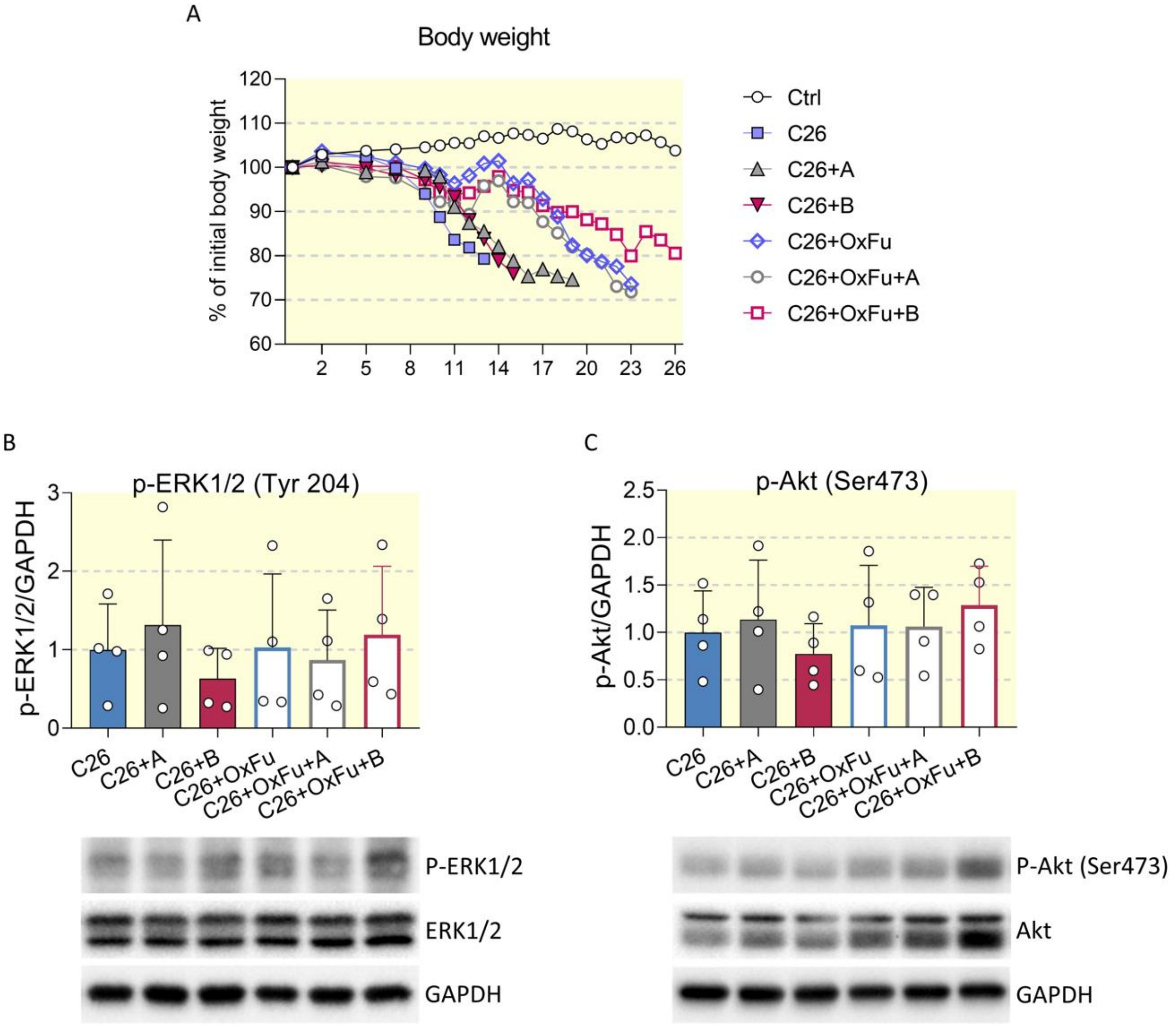

Figure S2. Time course of cancer cachexia and evaluation of ERK1/2 and Akt phosphorylation in C26-tumors. (A) Body weight change. N=5 animals per group. (B,C) Representative Western blot and densitometric analysis of cleaved caspase-3 in C26-tumors from C26-tumor bearing mice (C26), C26-tumor bearing mice treated with BCAA (C26+A) and C26-tumor bearing mice treated with BCAA+Di-Ala (C26+B). Three other groups received OxFu regimen (50 mg/kg 5-Fluorouracil and 6 mg/kg oxaliplatin, once a week per i.p.) as chemotherapy (C26+OxFu, C26+OxFu+A, C26+OxFu+B). GAPDH was chosen as loading control. N=4 mice. Data are represented as means±SD. Statistical analysis was performed by using one-way ANOVA followed by Tukey's post hoc test.

Supplementary Table 1: List of antibodies used in this study

**Antibody**	**Ref**	**Provider**	**Application and Dilution**
20S proteasome	sc- 374405	Santa Cruz Biotechnology	WB (1:1000)
Akt1	sc-5298	Santa Cruz Biotechnology	WB (1:1000)
AMPK	sc-25792	Santa Cruz Biotechnology	WB (1:1000)
Atrogin-1	ab168372	Abcam	WB (1:1000)
Beclin 1	sc-11427	Santa Cruz Biotechnology	WB (1:1000)
Cleaved Caspase-3	sc-56053	Santa Cruz Biotechnology	WB (1:300)
ERK 1/2	sc-94	Santa Cruz Biotechnology	WB (1:3000)
GAPDH	sc-32233	Santa Cruz Biotechnology	WB (1:5000)
Il-6	#12912	Cell Signaling Technology	WB (1:500)
LC3	L7543	Merck Life Science	WB (1:2000)
MuRF1	sc-398608	Santa Cruz Biotechnology	WB (1:1000)
Myostatin	sc-6885	Santa Cruz Biotechnology	WB (1:500)
NFkB p65	sc-372	Santa Cruz Biotechnology	WB (1:1000)
NFkB p-p65 (Ser536)	#3033	Cell Signaling Technology	WB (1:500)
p62	sc-48402	Santa Cruz Biotechnology	WB (1:1000)
p70S6K	sc-230	Santa Cruz Biotechnology	WB (1:100)
p-Akt1/2/3 (Ser473)	sc-7985	Santa Cruz Biotechnology	WB (1:500)
p-AMPK	sc-33524	Santa Cruz Biotechnology	WB (1:300)
p-ERK 1/2 (Tyr204)	sc-7383	Santa Cruz Biotechnology	WB (1:1000)
p-p70S6K (Thr389)	sc-4759	Santa Cruz Biotechnology	WB (1:200)
p-STAT3 (Tyr705)	#9145	Cell Signaling Technology	WB (1:500)
Puromycin (clone 12D10)	MABE343	Merck Life Science	WB (1:1000)

STAT3	sc-482	Santa Cruz Biotechnology	WB (1:1000)
Vinculin	sc-73614	Santa Cruz Biotechnology	WB (1:500)
Goat Anti-Mouse IgG (H + L)-HRP Conjugate	#1706516	Biorad Laboratories	WB (1:10000)
Goat Anti-Rabbit IgG (H + L)-HRP Conjugate	#1706515	Biorad Laboratories	WB (1:10000)

Supplementary Table 2: List of Primers used in quantitative RT-PCR analysis

**Gene**	**Sequence**
Cathepsin L	GTGGACTGTTCTCACGCTCAG<br>TCCGTCCTTCGCTTCATA GG
Gabarapl1	CATCGTGGAGAAGGCTCCTA<br>TCCTCAGGTCTCAGGTGGAT
Il-6	GCCAGAGTCCTTCAGAGAGA<br>TGGTCCTTAGCCACTCCTTC
MAFbx/Atrogin-1	CACATTCTCTCCTGGAAGGGC<br>TTGATAAAGTCTTGAGGGGAA
MuRF1	AGTGTCCATGTCTGGAGGT<br>AATGATGTTTTCCACCAGC
Gapdh	AACATCAAATGGGGTGAGGCC<br>GTTGTCATGGATGACCTTGGC
